# Nestin-Expressing Stem Cells Promote Nerve Growth in Long-Term 3-Dimensional Gelfoam®-Supported Histoculture

**DOI:** 10.1371/journal.pone.0067153

**Published:** 2013-06-19

**Authors:** Sumiyuki Mii, Fuminari Uehara, Shuya Yano, Benjamin Tran, Shinji Miwa, Yukihiro Hiroshima, Yasuyuki Amoh, Kensei Katsuoka, Robert M. Hoffman

**Affiliations:** 1 AntiCancer Inc., San Diego, California, United States of America; 2 Department of Surgery, University of California San Diego, San Diego, California, United States of America; 3 Department of Dermatology, Kitasato University School of Medicine, Kanagawa, Japan; Baylor College of Medicine, United States of America

## Abstract

We have previously reported that hair follicles contain multipotent stem cells which express nestin. The nestin-expressing cells form the hair follicle sensory nerve. In vitro, the nestin-expressing hair follicle cells can differentiate into neurons, Schwann cells, and other cell types. In the present study, the sciatic nerve was excised from transgenic mice in which the nestin promoter drives green fluorescent protein (ND-GFP mice). The ND-GFP cells of the sciatic nerve were also found to be multipotent as the ND-GFP cells in the hair follicle. When the ND-GFP cells in the mouse sciatic nerve cultured on Gelfoam® and were imaged by confocal microscopy, they were observed forming fibers extending the nerve. The fibers consisted of ND-GFP-expressing spindle cells, which co-expressed the neuron marker β-III tubulin, the immature Schwann-cell marker p75^NTR^ and TrkB which is associated with neurons. The fibers also contain nestin-negative spherical cells expressing GFAP, a Schwann-cell marker. The β-III tubulin-positive fibers had growth cones on their tips expressing F-actin, indicating they are growing axons. When the sciatic nerve from mice ubiquitously expressing red fluorescent protein (RFP) was co-cultured on Gelfoam® with the sciatic nerve from ND-GFP transgenic mice, the interaction of nerves was observed. Proliferating nestin-expressing cells in the injured sciatic nerve were also observed in vivo. Nestin-expressing cells were also observed in posterior nerves but not in the spinal cord itself, when placed in 3-D Gelfoam® culture. The results of the present report suggest a critical function of nestin-expressing cells in peripheral nerve growth and regeneration.

## Introduction

We previously demonstrated in 3-dimensional Gelfoam® histoculture that nestin-expressing cells in the whisker follicle bulge traffic to the truncated whisker sensory nerve and effect nerve growth and interaction with other nerves in vitro [1], [2].

We originally reported that the nestin-expressing stem cells are located in the permanent upper hair follicle in the bulge area of the hair follicle. The nestin-expressing cells have round/oval-shaped bodies with a typical diameter of 7 µm and two-three long elongated processes containing club-like bodies in the bulge area surround the hair shaft [3]–[5]. In vitro, the nestin-expressing hair follicle cells formed spheres and differentiated into neurons, glia, keratinocytes, smooth muscle cells, and melanocytes. The nestin-expressing cells in the spheres are positive for the stem cell marker CD34 [6].

When nestin-expressing cells from the mouse vibrissa bulge area or human scalp were implanted into the gap region of the severed sciatic nerve, they effected functional nerve repair. The transplanted nestin-expressing cells differentiated largely into Schwann cells, which are known to support neuron regrowth. The transplanted mice recovered the ability to walk normally [7], [8]. Nestin-expressing mouse vibrissa cells from the bulge area were also transplanted to the injury site of mice in which the thoracic spinal cord was severed. Most of the transplanted cells also differentiated into Schwann cells that effected repair of the severed spinal cord. The rejoined spinal cord recovered and extensive hind-limb locomotor performance was re-established [7], [8].

In the present study, we demonstrate that nestin-expressing cells in the sciatic nerve had the ability to form spheres and differentiate into neurons, glia, keratinocytes, and smooth muscle cells in vitro similar to hair follicle nestin-expressing cells. The nestin-expressing cells in sciatic nerves formed axon fibers to extend the nerve which can intermingle with other sciatic nerves in long-term 3-dimensional Gelfoam® histoculture.

## Materials and Methods

### Animals

Transgenic mice with nestin-driven GFP (ND-GFP) [3], [9], as well as red fluorescent protein DsRed2 (RFP) transgenic mice [10], [11], at different ages (4 weeks up to 5 months) (AntiCancer, Inc., San Diego, CA), were used to this study. All animal studies were conducted with an AntiCancer Institutional Animal Care and Use Committee (IACUC)-protocol specifically approved for this study and in accordance with the principals and procedures outlined in the National Institute of Health Guide for the Care and Use of Animals under Assurance Number A3873-1.

### Isolation of the sciatic nerve, dorsal root ganglion, and spinal cord

The mice were anesthetized with 30–50 µl ketamine solution (25 mg/ml) [1], [4]. In order to isolate the sciatic nerve, a skin incision was made in the medial side of the thigh of ND-GFP transgenic mice or RFP transgenic mice. The nerve was exposed between the short and long adductor muscles. Using an MZ6 binocular microscope (Leica, Wetzlar, Germany), the sciatic nerve was excised with fine forceps. The excised sciatic nerve was 3–4 mm long. In order to isolate the spinal cord, a skin incision was made on the dorsal side, and the spine between the 3rd and 8th thoracic vertebrae was exposed. The vertebral canal was opened after the spinous process, and the lamina were removed with a drill and the spinal cord was exposed. Using the binocular microscope, the spinal cord was excised with fine forceps. The isolated nerves and the spinal cord were washed in PBS three times before culture.

### Suspension culture of nestin-expressing cells of the sciatic nerve and sphere formation

The excised sciatic nerve was cut into small pieces, which were incubated in DMEM-F12 (GIBCO/BRL), containing B-27 (GIBCO/BRL), N2 (GIBCO/BRL), 1% penicillin and streptomycin (GIBCO/BRL) and 20 ng/ml basic fibroblast growth factor (Millipore) at 37°C, 5% CO2 100% humidity in 24-well tissue-culture dishes (Sarstedt). The medium was changed every other day. The nestin-expressing cells formed spheres by approximately 4 weeks in culture.

For differentiation, the colonies were centrifuged and the growth factor-containing DMEM-F12 medium was removed. The colonies were resuspended into fresh RPMI 1640 medium (GIBCO) containing 10% FBS. The colonies were cultured in four-well chamber slides (Fisher Scientific).

### Gelfoam® Histoculture of nerves

The excised sciatic nerve, dorsal root ganglion, and the spinal cord described above were put on sterile Gelfoam® (Pharmacia and Upjohn Co., Kalamazoo, MI) hydrated in cell culture medium. The sciatic nerves were arranged opposed to each other. Culture medium used was DMEM-F12 medium (GIBCO/BRL) containing B-27 (2.5%) (GIBCO/BRL), N2 (1%) (GIBCO/BRL) and 1% penicillin and streptomycin (GIBCO/BRL). The Gelfoam® cultures were incubated at 37°C, 5% CO2 100% humidity. The medium was changed every other day.

### Confocal laser-scanning microscopy

A confocal laser-scanning microscope (Fluoview FV1000, Olympus Corp., Tokyo, Japan) was used for two- (X,Y) and three-dimensional (3D, X,Y,Z) high-resolution imaging of nerves in histoculture. Fluorescence images were obtained using the 4x/0.10 Plan N, 10x/0.30 Plan-NEOFLUAR, 20x/0.50 UPlan FL N and 20x/1.00w XLUMplan FL objectives.

### Histology and Immunofluorescence staining

Tissues were fixed in pre-cooled 4% paraformaldehyde at room temperature (RT) for 2 hours and embedded in tissue freezing medium (Triangle Biomedical Science, Durham, NC) and frozen in nitrogen for 10 min at −80°C. Frozen sections of 7–10 µm thickness were prepared with a CM1850 cryostat (Leica). The frozen sections were washed with PBS three times. Some frozen sections were processed for hematoxilin and eosin staining. Immunofluoresence staining procedures had the following steps: (1) 5% normal goat serum was applied at RT for 1 hour. (2) Primary antibodies were applied at RT for 2 hours. The primary antibodies used were anti-β III tubulin mAb (mouse, 1∶100, Santa Cruz); anti-glial fibrillary acidic protein (GFAP) mAb (mouse, 1∶250, BD Pharmingen); anti-S100 mAb (mouse, 1∶200, Millipore); anti-p75^NTR^ mAb (rabbit, 1∶3200, Cell Signaling); anti-TrkA mAb (rabbit, 1∶50, Santa Cruz) and anti-TrkB mAb (rabbit, 1∶50, Santa Cruz). (3) Secondary antibodies used were: goat anti-mouse IgG Alexa Fluor® 555 (1∶1000, Cell Signaling); goat anti-rabbit IgG (H+L) Alexa Fluor® 555 (1∶1000, Cell Signaling), RT, dark, 1 hour. (4) Alexa Fluor® 647 Phalloidin (1∶40, invitrogen) for F-actin detection, RT, dark, 1 hour. (5) DAPI (1∶48000, invitrogen), RT, dark, 3 minutes. (6) Slides were mounted with Fluoromount (Sigma) and observed under confocal laser-scanning microscopy. All findings of immunofluorescence staining were compared with positive and negative controls.

### In vivo imaging of the injured sciatic nerve of ND-GFP transgenic mice

ND-GFP transgenic mice were anesthetized with 30–50 µl ketamine solution (25 mg/ml). A skin incision was made in the medial side of the thigh of ND-GFP transgenic mice. The nerve was exposed between the short and long adductor muscles. Using the MZ6 binocular microscope, the sciatic nerve was cut with fine forceps. The transected nerve was sutured with a 9-0 suture (Ethibond extra polyester suture, Ethicon). The skin incision was subsequently closed with a 6-0 suture (Ethibond extra polyester suture, Ethicon). All surgical procedures were performed under sterile conditions. The sutured sciatic nerve was removed one week later and observed under fluorescence microscopy (MVX-10, Olympus Corp., Tokyo, Japan).

## Results

### Differentiation potential of nestin-expressing spheres cultured from the sciatic nerve

**Figure 1:**
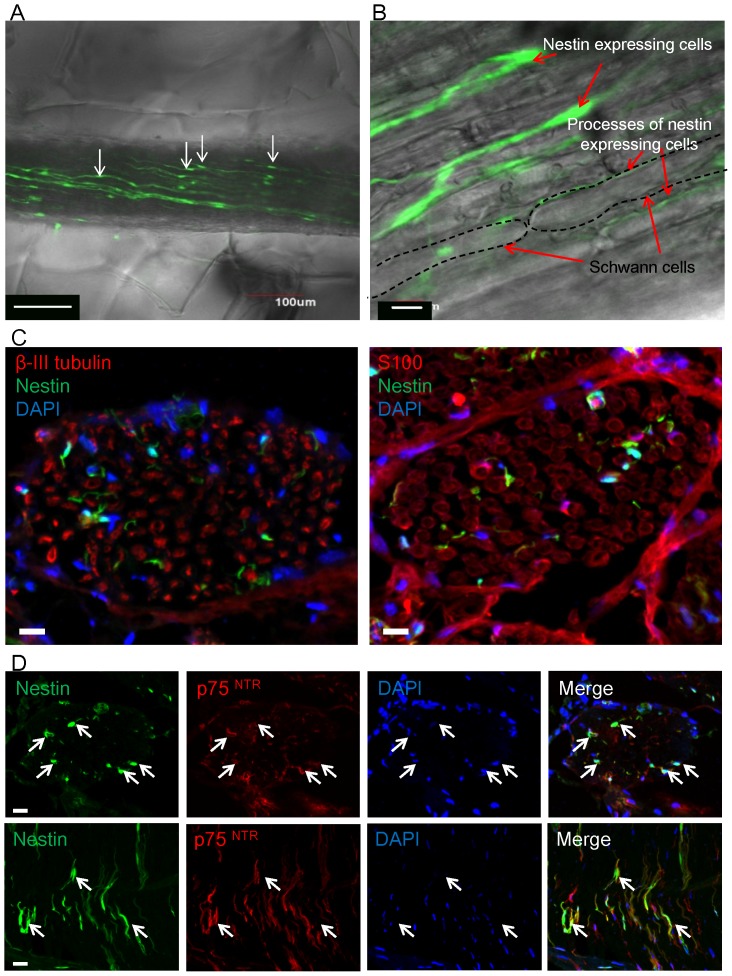
Location and characteristics of ND-GFP-expressing cells in the sciatic nerve. (A) The first day of Gelfoam® histoculture of a sciatic nerve bundle removed from the ND-GFP transgenic mouse. The sciatic nerve contained ND-GFP-expressing cells which had long processes (white arrows). Bar: 100 µm. (B) High-magnification image of the sciatic nerve showed that ND-GFP expressing cells and their processes are located between Schwann cells. Bar: 10 µm. (C) Transverse sections of the sciatic nerve of the ND-GFP mouse. The ND-GFP-expressing cells did not express β-III tubulin and S100. Bar: 10 µm. (D) Transverse and longitudinal sections of the sciatic nerve of the ND-GFP mouse. The ND-GFP-expressing cells (white arrows) co-expressed p75^NTR^. Bar: 10 µm.

The nestin-expressing cells within the sciatic nerve isolated form ND-GFP mice had oval-shaped bodies with a typical diameter of 10 µm and two long elongated processes placed along the nerve fiber (Fig. 1A, B) The ND-GFP-expressing nerve cells co-expressed p75^NTR^ a neural-crest and immature Schwann-cell marker (Fig. 1D), but were negative for S100, a Schwann cell marker, and β-III tubulin, a neuron marker. Additionally, CD34, a stem cell marker, was negative.

**Figure 2:**
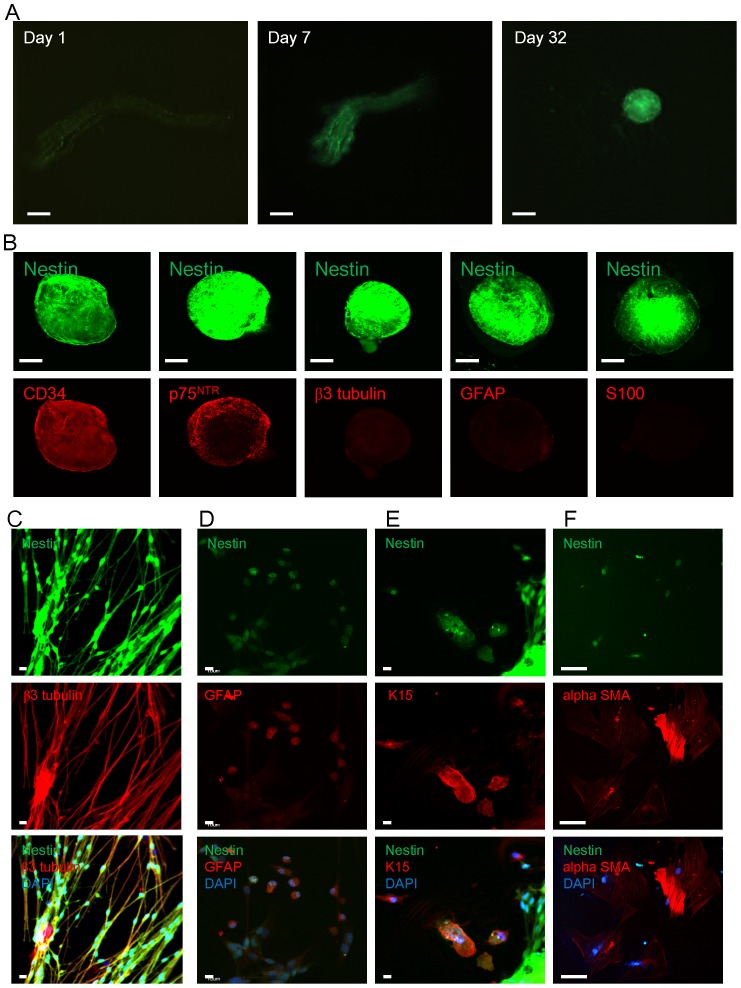
Sphere formation and differentiation from the sciatic nerve in suspension culture. (A) The sciatic nerve was cultured in DMEM-F12 medium containing bFGF. The ND-GFP-expressing cells proliferated and formed spheres by day 32. Bar: 100 µm. (B) The spheres expressing ND-GFP co-expressed p75^NTR^ and CD34 but did not express β-III tubulin S100 and GFAP. Bar: 100 µm. (C) The ND-GFP-expressing spheres were switched to RPMI 1640 medium containing 10% FBS from DMEM-F12 containing B-27, N2 and bFGF and began to differentiate. At 7 days after switching into medium containing FBS, β-III tubulin-positive neuronal cells which expressed ND-GFP were observed. Bar: 10 µm. (D) At 14 days, the ND-GFP-expressing cells differentiated to GFAP-positive glial cells. Bar: 10 µm (E) At 7 days after culture in FBS medium, the ND-GFP-expressing cells differentiated to K15-positive keratinocytes, some of which still expressed nestin. Bar: 10 µm. (F) At 30 days after culture in FBS medium, the ND-GFP-expressing cells differentiated to α-SMA-positive smooth muscle cells. Bar: 50 µm.

The sciatic nerves containing ND-GFP expressing cells were cultured in DMEM-F12 medium with bFGF. The ND-GFP expressing cells proliferated within the nerve. Four to 8 weeks after initiation of culture, the ND-GFP expressing cells formed spheres (Fig. 2A). The spheres expressed ND-GFP, co-expressed p75^NTR^ and CD34, even though they did not express CD34 before culture. The ND-GFP positive spheres did not express β III tubulin, GFAP, or S100 (Fig. 2B).

The ND-GFP-expressing spheres were switched to RPMI 1640 medium containing 10% FBS from DMEM-F12 containing B-27, N2, and bFGF which had kept the ND-GFP expressing cells in the spheres in an undifferentiated state. Then, they began differentiation. At 7 days after switching to RPMI 1640 medium containing FBS, β-III tubulin positive neuronal cells which maintain ND-GFP expression were observed. At 14 days after medium change, the ND-GFP expressing cells differentiated to GFAP-positive glial cells (Fig. 2D). At 5 days after culture in the FBS medium, the ND-GFP expressing cells differentiated to K15-positive keratinocytes. Some K15-positive cells still expressed nestin (Fig. 2E). At 4 weeks of culture in FBS medium, the ND-GFP expressing cells differentiated to α-SMA-positive smooth muscle cells (Fig. 2F).

### Nestin-expressing cells participate in nerve growth in 3D Gelfoam® culture

**Figure 3:**
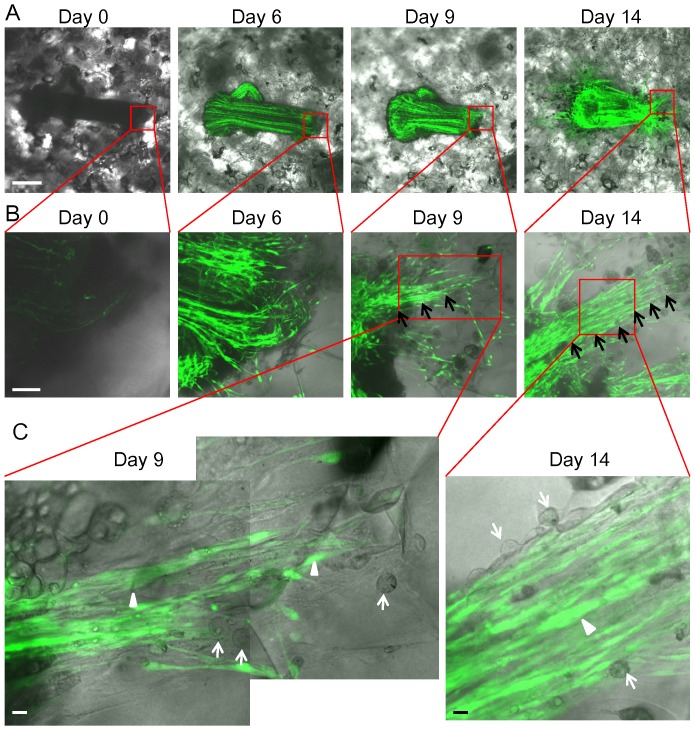
ND-GFP-expressing cells proliferated in the sciatic nerve and formed fibers in 3D Gelfoam® histoculture. (A) Time-course imaging of ND-GFP-expressing cell trafficking in the histocultured sciatic nerve from an ND-GFP mouse. The nerve became enriched with ND-GFP-expressing cells by day 14. At day 6, the ND-GFP-expressing cells proliferated in extending fibers and migrated into the Gelfoam®. At day 14, many fibers consisting of ND-GFP-expressing cells extended widely and radially around the nerve. Bar: 500 µm. (B) Magnified images of the growing nerve. At day 6, the ND-GFP-expressing cells proliferated as fibers and migrated into the Gelfoam®. Bar: 100 µm. (C) High-magnification images of the area inside the boxes in Figure 3B. At day 9, the ND-GFP-expressing cells grew in fibers (white arrow heads). Some spherical cells proliferated in the growing fibers. They did not express nestin (white arrows). At day 14, the spindle cells expressed ND-GFP (white arrow head). In addition, there were spherical cells without nestin expression (white arrow). Bar: 10 µm.

The sciatic nerve became enriched with ND-GFP-expressing cells in Gelfoam® histoculture. High-magnification images show that the ND-GFP-expressing cells proliferated, forming fibers extending into the Gelfoam® (Fig. 3). Higher-magnification images of the growing fibers demonstrated the presence of both spindle and spherical cells. The spindle cells highly expressed ND-GFP (Fig. 3). The fibers continued to extend at day 28 in Gelfoam® culture.

**Figure 4:**
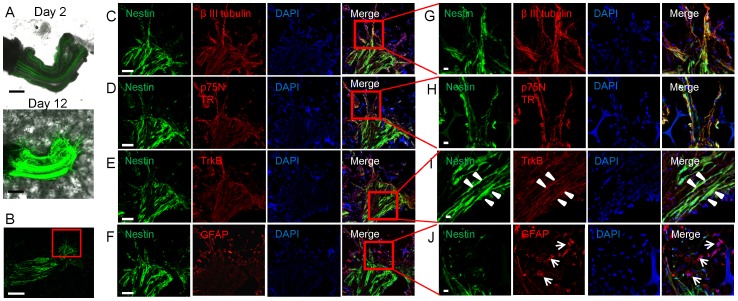
Nestin-positive spindle cells expressed neuronal markers and nestin-negative spherical cells expressed glial marker in growing nerve fibers in 3D Gelfoam® histoculture. (A) A sciatic nerve was cultured on Gelfoam®. At day 12, the sciatic nerve was enriched with ND-GFP-expressing cells and many fibers consisting of ND-GFP-expressing cells extended from the growing nerve. Bar: 500 µm. (B) One section from Figure 5A on day 12. Bar: 500 µm. (C-F) Figures 5C-F are magnified images from the box in Figure 5B. ND-GFP-expressing cells in the sciatic nerve co-expressed β-III tubulin, p75^NTR^ and TrkB. Bar: 100 µm. (G, H) Figures 5G, H are magnified images from the box in Figure 5C, D. There were fibers extending from the nerve. These fibers consisted of ND-GFP-expressing spindle cells. The spindle cells expressing ND-GFP co-expressed β-III tubulin and p75^NTR^. Bar: 10 µm. (I) High-magnification images of the area inside the box in Figure 5E show that the spindle cell, expressed ND-GFP in the nerve and co-expressed TrkB. Bar: 10 µm. (J) Magnified images from the area inside the box in Figure 5F. Spherical cells were observed in the growing nerve. Most of the spherical cells expressed GFAP but not ND-GFP. Bar: 10 µm.

The ND-GFP-expressing cells extending the nerve in Gelfoam® co-expressed p75^NTR^, TrkB, and β-III tubulin (Fig. 4). High-magnification images show that spindle cells expressed ND-GFP, co-expressed p75^NTR^, TrkB. and β-III tubulin. (Fig. 4G-I). Most of the many spherical cells expressed GFAP but not nestin (Fig. 4F,J).

### Fibers extending the nerve in 3D Gelfoam® histoculture express β-III tubulin, and their tips express phalloidin-positive F-actin

**Figure 5:**
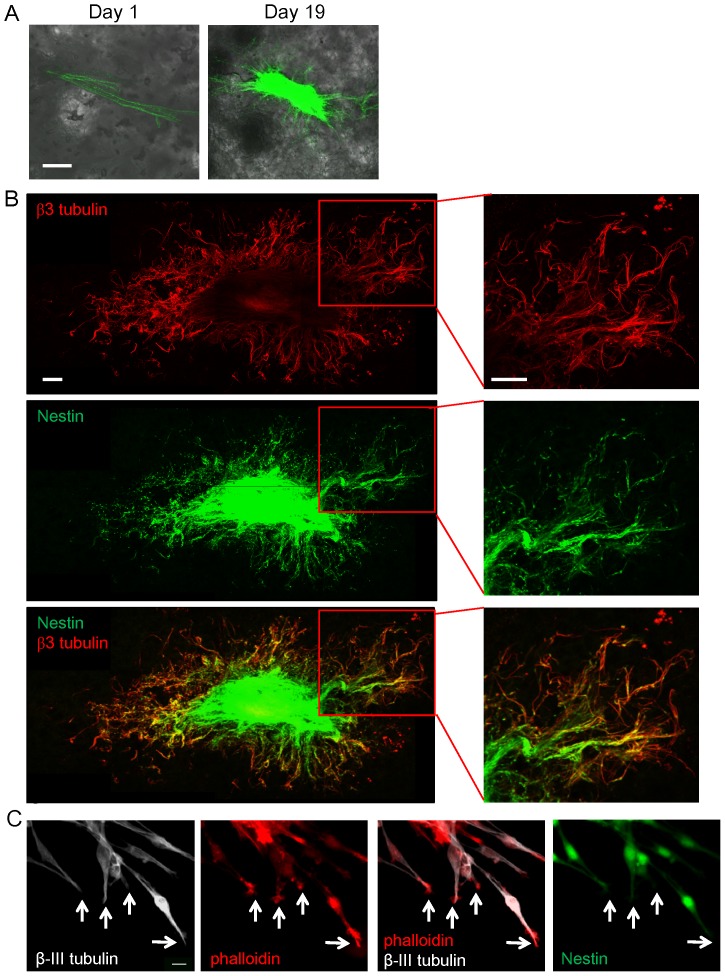
Fibers growing from the sciatic nerve in 3D Gelfoam® histoculture expressed β-III tubulin and contained tips expressing phalloidin-positive F-actin. (A) A sciatic nerve from an ND-GFP mouse was cultured for 19 days on Gelfoam®. The nerve became enriched with ND-GFP-expressing cells and many fibers extended from the growing nerve. Bar: 500 µm. (B) Immunofluorescence stained images of β-III tubulin (red) demonstrated that many β-III tubulin-positive fibers extended from the nerve stump and extended widely and radially around the nerve. Merged images of Figure 5 demonstrated that β-III tubulin positive fibers consisted of ND-GFP-expressing cells. Bar: 500 µm. (C) Tips of the β-III tubulin (white)-positive fibers had phalloidin (red)-positive F-actin. The presence of F-actin indicates that tips are axons growth cones. White arrows: tips of the β-III tubulin-positive fiber. Bar: 10 µm.

Immunofluorescence demonstrated that many β-III tubulin-positive fibers extended widely and radially around the nerve. The fibers consisted of ND-GFP expressing cells (Fig. 5). The tips of the β-III tubulin-positive fibers expressed phalloidin-positive F-actin. These results suggested that β-III tubulin-positive fibers are axons growing from the sciatic nerve (Fig. 5).

### Inter-nerve co-mingling in 3D Gelfoam® histoculture

**Figure 6:**
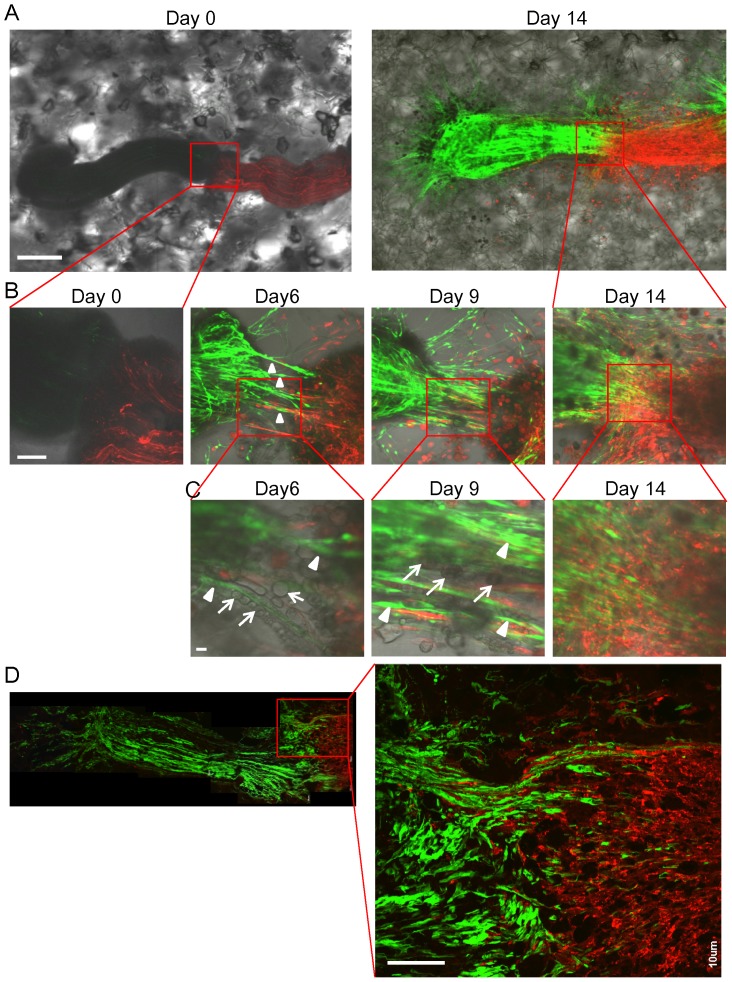
Intermingling of growing sciatic nerve in 3D Gelfoam® histoculture. (A) A sciatic nerve from a ND-GFP transgenic mouse was placed on Gelfoam® next to the sciatic nerve from an RFP transgenic mouse. At day 14, the sciatic nerve from the ND-GFP mouse was enriched with ND-GFP-expressing cells and intermingled with to the RFP-expressing sciatic nerve. Bar: 500 µm. (B) Magnified images of the area inside the box in Figure 6A show that the ND-GFP-expressing cells proliferated in fibers growing from the nerve extending toward the other sciatic nerve. At day 9, the thickest fibers appeared between both sciatic nerves. At day 14, the two nerves intermingling with each other. Bar: 100 µm. (C) Magnified images of the area inside the box in Figure 6B show that the fibers consisted of ND-GFP-expressing spindle cells (white arrow heads) and ND-GFP-negative spherical cells (white arrows). The spherical cells formed a line between both sciatic nerves and ND-GFP-expressing spindle cells extended among the lines. Bar: 10 µm. (D) A section of the intermingling two nerves. High-magnification images show that ND-GFP-expressing fibers growing from the sciatic nerve from the ND-GFP mouse invaded deeply into the RFP-expressing sciatic nerve. Bar: 100 µm.

The sciatic nerve obtained from ND-GFP transgenic mice was co-cultured on Gelfoam® with a sciatic nerve from RFP transgenic mice (Fig. 6A). The ND-GFP-expressing fibers of the sciatic nerve intermingled with the fibers of the co-cultured RFP-expressing sciatic nerve at day 14 of histoculture (Fig. 6A, B). The ND-GFP-expressing cells proliferated, forming radial fibers which extended the nerve. The fibers consisted of ND-GFP-expressing spindle cells and ND-GFP-negative spherical cells (Fig. 6C). ND-GFP-expressing fibers invaded deeply into the RFP-expressing sciatic nerve (Fig. 6D).

### Interaction of the extending sciatic nerve and the dorsal root ganglion in 3D Gelfoam® histoculture

**Figure 7:**
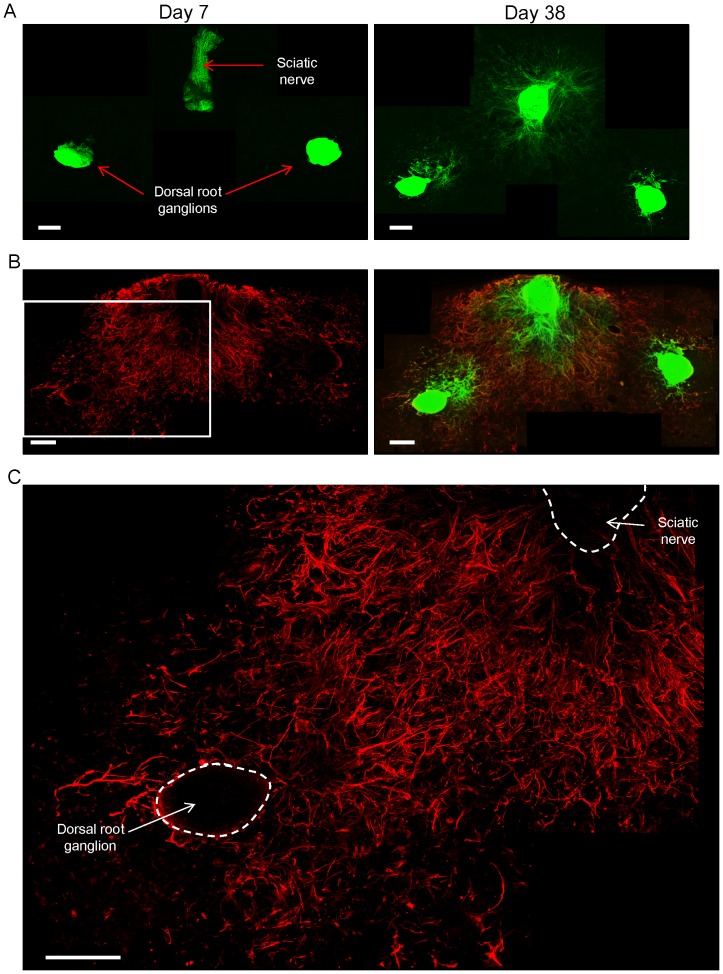
The growing sciatic nerve intermingled with the dorsal root ganglion in 3D Gelfoam® histoculture. (A) A sciatic nerve was placed in Gelfoam® histoculture next to a dorsal root ganglion, both from a ND-GFP mouse. At day 38, many ND-GFP-expressing fibers were seen extending from both the sciatic nerve and co-cultured dorsal root ganglion. Bar: 500 µm. (B) Immunofluorescence staining of β-III tubulin (red) demonstrated that many β-III tubulin-positive fibers extended from both the sciatic nerve and the dorsal root ganglions. The fibers consisted of ND-GFP expressing cells. Bar: 500 µm. (C) Magnified image of the area inside the box in Figure 7B shows that many β-III tubulin-positive fibers extended widely and radially both from the sciatic nerve and the dorsal root ganglion. β-III tubulin-positive fibers from both nerves intermingled with each other. Bar: 500 µm.

The sciatic nerve was co-cultured on Gelfoam® with a dorsal root ganglion, both isolated from an ND-GFP transgenic mouse (Fig. 7A). Many GFP-expressing fibers were imaged extending from both the sciatic nerve and the co-cultured dorsal root ganglion at day 38 of histoculture (Fig. 7A). Immunofluorescence staining also demonstrated that many β-III tubulin-positive fibers extended from both the dorsal root ganglion and the sciatic nerve. The fibers consisted of ND-GFP-expressing cells (Fig. 7B). β-III tubulin-positive fibers extending from both the sciatic nerve and the dorsal root ganglion intermingled each other (Fig. 7C).

### ND-GFP-expressing cells are located in peripheral dorsal root nerves but not in the spinal cord in 3D Gelfoam® histoculture

**Figure 8:**
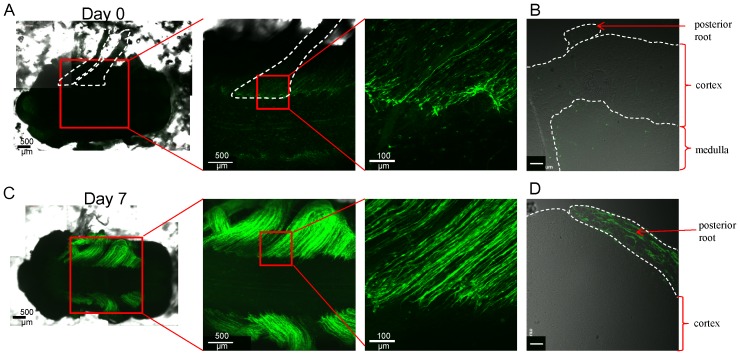
ND-GFP-expressing cells are located in dorsal peripheral nerve roots but not in the spinal cord in 3D Gelfoam® histoculture. (A) A spinal cord with posterior roots removed from an ND-GFP transgenic mouse was put in Gelfoam® histoculture. On day 0, some ND-GFP-expressing cells were observed in the origin of posterior root (enclosed by white dashed lines). (B) Cross section of the spinal cord just after removal show that there were a few ND-GFP-expressing cells in the posterior root. White bar: 50 µm. (C) At day 7, posterior roots were enriched with ND-GFP-expressing cells. (D) Cross section of the spinal cord cultured for 7 days show that ND-GFP-expressing cells proliferated in the posterior root but not in the cortex of spinal cord. White bar: 50 µm.

The spinal cord with posterior roots, excised from an ND-GFP transgenic mouse, was put in Gelfoam® histoculture. On day 0, some ND-GFP-expressing cells were seen in the origin of posterior roots (Fig. 8A,B). At day 7, the posterior roots were enriched with ND-GFP-expressing cells but were not observed in the cortex of the spinal cord (Fig. 8C,D).

### The injured sciatic nerve contained ND-GFP expressing cells in vivo

**Figure 9:**
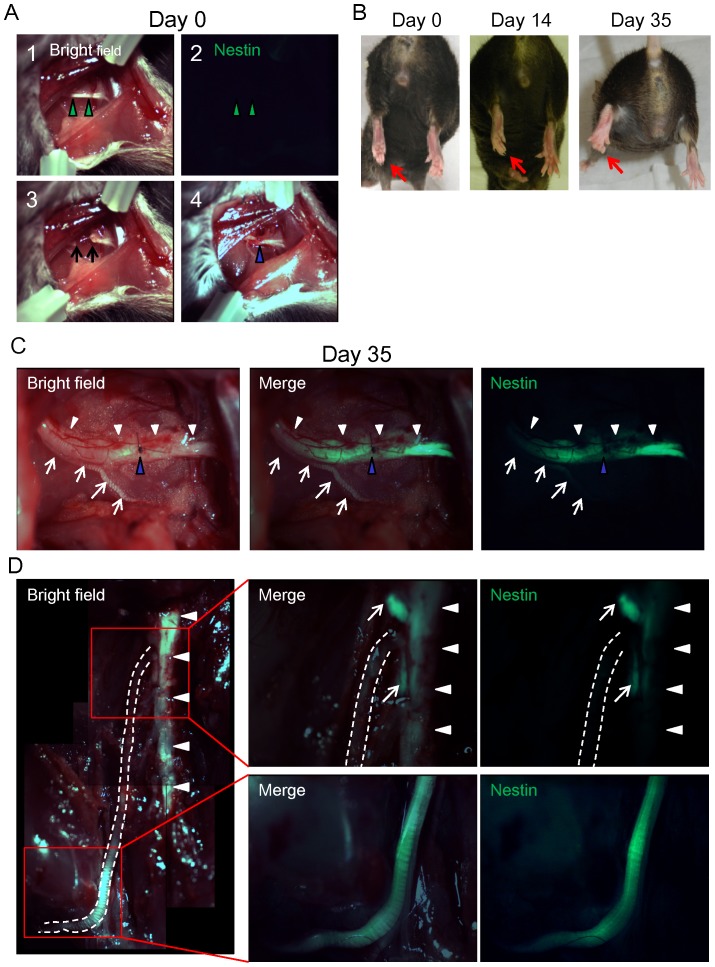
The injured sciatic nerve expressed ND-GFP in vivo. (A) 1: The left sciatic nerve (green arrow heads) was exposed between the short and long adductor muscles. 2: ND-GFP expression was not observed. 3: The sciatic nerve was severed (black arrows: the nerve stumps). 4: The transected nerve was rejoined with a 9-0 suture (blue arrow head). (B) At day 0, left plantar (red arrows) was paralyzed. At day 35, the left plantar could show digit flection. (C) At day 35, the injured nerves (white arrow heads) connected (blue arrow head: point of suture). Strong nestin expression was observed in the injured sciatic nerve. Note that the intact nerve (white arrows) did not express ND-GFP. (D) The injured sciatic nerve (white dashed line) expressed ND-GFP. Nestin expression was stronger on the distal side compared to the proximal side of the sciatic nerve and spine (white arrow heads). White arrows: dorsal root ganglions.

The sciatic nerve was severed in an ND-GFP mouse and then the transected nerve was sutured, and the wound was closed (Fig. 9A). By day 35, the injured nerve reconnected and strong nestin expression was observed in the injured sciatic nerve (Fig. 9C) Nestin expression was stronger on the distal side compared to the proximal side of the sciatic nerve and spine (Fig. 9D).

## Discussion

The present study imaged the presence and behavior of ND-GFP-expressing cells in nerves in Gelfoam® histoculture, using confocal microscopy. The ND-GFP-expressing cells in the histocultured sciatic nerve initially expressed p75^NTR^, a neural crest cell marker, but not S100, a Schwann cell marker, and β-III tubulin, a neutron marker. The nestin-expressing cells formed spheres in suspension culture in DMEM-F12 medium with bFGF. This medium kept the ND-GFP expressing cells in the spheres in an undifferentiated state. It is possible that EGF could replace the bFGF and this will be tested in future experiments. The spheres consisted of nestin-expressing cells that co-expressed p75NTR and developed expression of the stem cell marker CD34, although they did not express CD34 in the sciatic nerve immediately after removal from the ND-GFP transgenic mouse. Nestin, p75^NTR^, and CD34 positivity indicated the sphere cells were in a relatively undifferentiated state.

There are reports on the presence of neural stem cells in peripheral nerves. Widera et al. [12] reported that neurospheres formed from the sciatic nerve expressed nestin, p75^NTR^, and SOX-2, consistent with our results. Takagi et al. [13] also reported neurospheres formed from the injured sciatic nerve expressed nestin, p75^NTR^. They could obtain more neurospheres from the injured sciatic nerve compared to the intact sciatic nerve. This result coincides with our observation that the injured sciatic nerve showed strong nestin expression in vivo.

In our study, the nestin-expressing cells of the spheres derived from the sciatic nerve could readily differentiate into β-III tubulin-positive neurons, GFAP-positive glial cells, K15-positive keratinocytes, and at α-SMA-positive smooth muscle cells after transfer to RPMI 1640 medium with 10% FBS. Widera et al. reported that neurospheres derived from the sciatic nerve differentiated into ectodermal, mesodermal, and endodermal linages [12].

The enrichment and proliferation of the ND-GFP-expressing cells in the sciatic nerve and their organization into fibers enabled the sciatic nerve to elongate in Gelfoam® histoculture. The growing fibers could intermingle with a co-cultured sciatic nerve and the dorsal root ganglion in Gelfoam® histoculture. The fibers of the sciatic nerve grew toward and intermingled with the other sciatic nerve or nerve ganglion. Fibers extending from the elongating sciatic nerve contained spindle cells expressing ND-GFP and β-III tubulin, features of axons [13]. The ND-GFP-expressing spindle cells also co-expressed p75^NTR^ and TrkB, which are receptors of BDNF, both of which are expressed mainly by neurons. The fibers also expressed F-actin on their tips detected by phalloidin staining, further suggesting the fibers are growing axons. The ND-GFP-expressing cells also proliferated in the injured sciatic nerve in vivo.

It is widely known that peripheral nerve injury can recover much easier than central nerve injury. This report demonstrated that peripheral nerves contain abundant nestin-expressing cells in contrast to the spinal cord [14].

There have been numerous studies on explanted nerves and peripheral nerve recovery in vitro from the proximal side but only a few are on the distal side [15]–[22]. In the present study, we observed growing axons from the distal side of an injured peripheral nerve in Gelfoam® histoculture. We also observed that the elongating sciatic nerve can intermingle with other nerves in Gelfoam® histoculture.

The results of the present report suggest that Gelfoam® 3D culture is a physiologic system to support nerve growth and intermingling and should have broad application.

The present study suggests a major role for the nestin-expressing cells is the extension of the sciatic nerve. The observations in the present report should enable future studies of the elongation and joining of many types of peripheral nerves to improve this process for clinical application.
